# APPIAN: Automated Pipeline for PET Image Analysis

**DOI:** 10.3389/fninf.2018.00064

**Published:** 2018-09-26

**Authors:** Thomas Funck, Kevin Larcher, Paule-Joanne Toussaint, Alan C. Evans, Alexander Thiel

**Affiliations:** ^1^Montreal Neurological Institute, McGill University, Montreal, QC, Canada; ^2^Jewish General Hospital and Lady Davis Institute for Medical Research, Montreal, QC, Canada; ^3^Biospective, Inc., Montreal, QC, Canada; ^4^Department of Neurology and Neurosurgery, McGill University, Montreal, QC, Canada

**Keywords:** open science, automation, pipeline, software, quality control, PET

## Abstract

APPIAN is an automated pipeline for user-friendly and reproducible analysis of positron emission tomography (PET) images with the aim of automating all processing steps up to the statistical analysis of measures derived from the final output images. The three primary processing steps are coregistration of PET images to T1-weighted magnetic resonance (MR) images, partial-volume correction (PVC), and quantification with tracer kinetic modeling. While there are alternate open-source PET pipelines, none offers all of the features necessary for making automated PET analysis as reliably, flexibly and easily extendible as possible. To this end, a novel method for automated quality control (QC) has been designed to facilitate reliable, reproducible research by helping users verify that each processing stage has been performed as expected. Additionally, a web browser-based GUI has been implemented to allow both the 3D visualization of the output images, as well as plots describing the quantitative results of the analyses performed by the pipeline. APPIAN also uses flexible region of interest (ROI) definition—with both volumetric and, optionally, surface-based ROI—to allow users to analyze data from a wide variety of experimental paradigms, e.g., longitudinal lesion studies, large cross-sectional population studies, multi-factorial experimental designs, etc. Finally, APPIAN is designed to be modular so that users can easily test new algorithms for PVC or quantification or add entirely new analyses to the basic pipeline. We validate the accuracy of APPIAN against the Monte-Carlo simulated SORTEO database and show that, after PVC, APPIAN recovers radiotracer concentrations within 93–100% accuracy.

## Introduction

**FIGURE 1:**
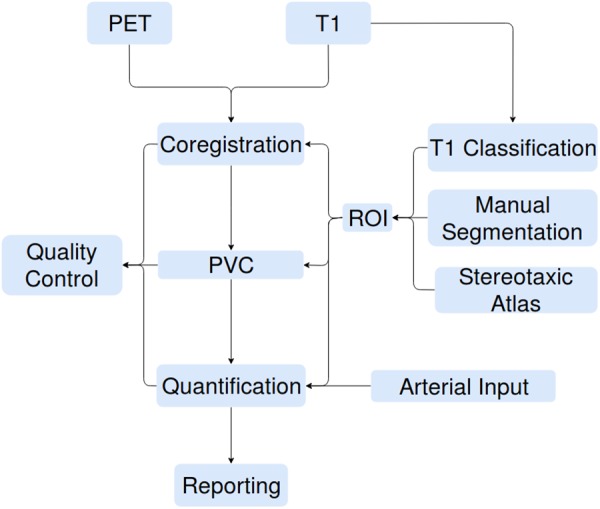
APPIAN performs all processing steps necessary to obtain quantitative parameters from reconstructed PET images. Flexible definition of ROI allows use of APPIAN for a wide variety of experimental designs. Integrated QC helps ensure that the pipeline performs as expected.

The increasing availability of large brain imaging data sets makes automated analysis essential. Not only is automated analysis important for saving time, but it also increases the reproducibility of research. No existing post-reconstruction positron emission tomography (PET) software package satisfies all the needs of researchers, specifically code that is free, open-source, language agnostic, easily extendible, deployable on web platforms as well as locally, and including all necessary processing steps prior to statistical analysis. We therefore present APPIAN (Automated Pipeline for PET Image Analysis) a new open-source pipeline based on NiPype (Gorgolewski et al., 2011) for performing automated PET data analysis. The starting point for APPIAN are reconstructed PET images on which all necessary processing steps are performed to obtain quantitative measures from the original PET images (**Figure 1**). In conjunction with the reconstructed PET image, APPIAN uses T1-weighted MR images to define regions of interest (ROI) that are used at multiple processing stages. Briefly, APPIAN (1) coregisters the T1 MR image with the PET image, (2) defines ROI necessary for later processing steps, (3) performs partial-volume correction (PVC), (4) calculates quantitative parameters, (5) produces a report of the results, and finally, (6) performs QC on the results (see **Figure 1** for a schema of APPIAN, and Discussion section for a detailed description of the pipeline, complete with flowchart).

## Materials and Methods

### Pipeline Overview

#### Coregistration

Positron emission tomography images are coregistered to the corresponding non-uniformity corrected (Sled et al., 1998) T1 MR-images using a six parameter linear fitting algorithm that minimizes normalized mutual information. The algorithm is based on minctracc^1^ and proceeds hierarchically by performing iterative coregistration at progressively finer spatial scales (Collins et al., 1994). Coregistration is performed in two stages, the first using a binary mask for the PET and the T1 MR images, respectively, to obtain a coarse coregistration. This is followed by a second registration step to refine the initial fit between the PET and T1 MR images without the use of the binary images.

#### MR Image Processing

T1 structural preprocessing is performed if the user does not provide a binary brain mask volume and a transformation file that maps the T1 MR image into stereotaxic space. If these inputs are not provided, APPIAN will automatically coregister the T1 MR image to stereotaxic space. By default, the stereotaxic space is defined on the ICBM 152 6th generation non-linear brain atlas (Mazziotta et al., 2001), but users can provide their own stereotaxic template if desired. Coregistration is performed using an iterative implementation of minctracc (Collins et al., 1994). Brain tissue extraction is performed in stereotaxic space using BEaST (Eskildsen et al., 2012). In addition, tissue segmentation can also be performed on the normalized T1 MR image. Currently, only ANTs Atropos package (Avants et al., 2011) has been implemented for T1 tissue segmentation but this can be extended based on user needs.

#### Regions of Interest

Regions of interest have an important role in three of the processing steps in APPIAN: PVC, quantification, and reporting of results. ROIs are used in PVC algorithms to define anatomical constraints. When no arterial input is available for quantification, a reference ROI is placed in a brain region devoid of specific tracer binding. Finally, when reporting results from APPIAN, ROIs are needed to define the brain areas from which average parameters are calculated for final statistical analysis. ROIs for each of these processing steps can be defined from one of three sources. The simplest ROI are those derived from a classification of the T1 MR image, e.g., using ANIMAL (Mazziotta et al., 2001), prior to using APPIAN. Users can also use tissue classification software implemented in APPIAN to classify their T1 MR images, thereby eliminating the need to run a strictly MR image-based pipeline prior to using APPIAN.

Regions of interest can also be defined on a stereotaxic atlas, e.g., AAL (Tzourio-Mazoyer et al., 2002), with a corresponding template image. In this case, the template image is non-linearly coregistered to the T1 MR image in native space, and subsequently aligned to the native PET space of the subject. Finally, it is frequently necessary to manually define ROI on each individual MR image, for instance when segmenting focal brain pathologies such as a tumor or ischemic infarct. This option is also implemented in APPIAN.

#### Partial-Volume Correction

In PET, partial-volume effects result from the presence of multiple tissue types within a single voxel and the blurring of the true radiotracer concentrations. PVC of PET images is thus necessary to accurately recover the true radiotracer distribution and, for example, differentiate between true neuronal loss from cortical thinning. Several methods have been proposed to perform PVC, many of which are implemented in PETPVC (Thomas et al., 2016). In addition, we have also implemented idSURF (Funck et al., 2014), a voxel-wise iterative deconvolution that uses anatomically constrained smoothing to control for noise amplification while limiting the amount of spill-over between distinct anatomical regions. APPIAN thus allows the user to select the appropriate PVC method based on their needs and their data. If the desired PVC method is not implemented in APPIAN, it can be easily included in the pipeline by creating a file describing the inputs and outputs of the method.

#### Quantification

In PET images, quantitative biological or physiological parameters—such as non-displaceable binding potential or cerebral blood flow—are often calculated from the measured temporal change of tissue radiotracer concentration, so-called time activity curves (TACs), within voxels or ROIs. Many models exist for performing quantification depending on the type of radiotracer, parameter of interest, and time frames acquired. The quantification methods available in APPIAN are from the Turku PET Centre tools (Oikonen, 2017). Currently, the implemented models are: the Logan Plot (Logan et al., 1990), Patlak–Gjedde Plot (Gjedde, 1982; Patlak et al., 1983), Simplified Reference Tissue Model (Gunn et al., 1997), and standardized uptake value (Sokoloff et al., 1977). APPIAN implements both voxel-based and ROI-based quantification methods. It can also process arterial input functions as well as input functions from reference regions devoid of specific binding. Arterial inputs are in the “.dft” format described by the Turku PET Centre^2^.

#### Results Report

The ROI defined in “MR Image Processing” section are used to calculate regional mean values for the parameter of interest from the output images after coregistration, PVC and quantification processing steps. Additionally, if cortical surface meshes are provided by the user, the output images can be interpolated on these meshes and be used to derive surface-based parameter estimates. Regional mean parameter values are saved in wide format ‘.csv’ files in the so-called ‘vertical format’ (i.e., the output measure from each subject and each region is saved in a single column). This standardized data format simplifies subsequent analysis with statistical software, such as R (R Core Team, 2016) or scikit-learn (Pedregosa et al., 2001).

APPIAN also calculates group-level descriptive statistics obtained from the output images. The group-level statistics that are provided exploit the BIDS naming convention which requires that file names include the subject ID, the task or condition, and the scanning session. APPIAN thus provides users with summary statistics for the subjects, tasks, and sessions. Descriptive statistics are plotted and displayed in a web browser-based GUI to allow simple and easy visualization of the results.

#### Quality Control and Visualization

**FIGURE 2:**
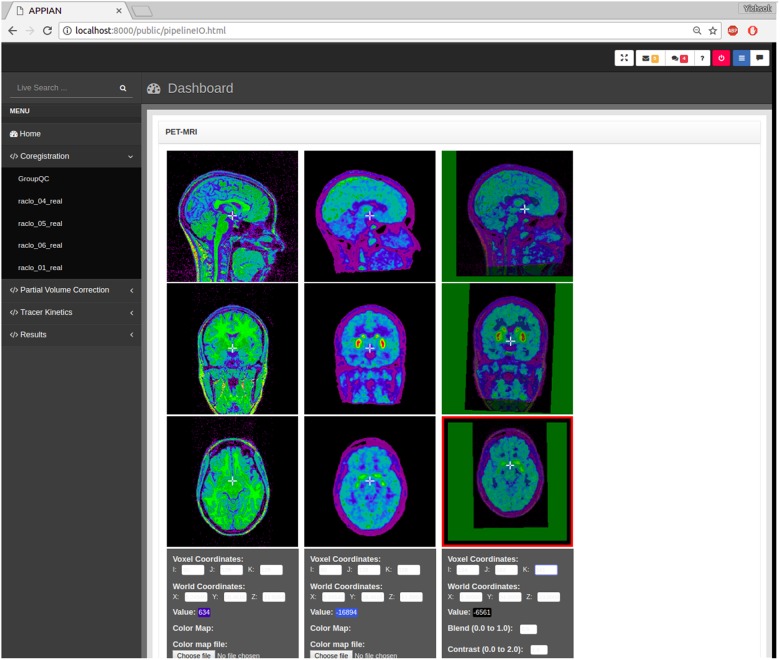
Output images produced by APPIAN can be viewed via a web browser-based dashboard. Visual QC for the coregistration stage can be performed by viewing the MRI, PET, and the fusion images of the two.

APPIAN includes both visual and automated quality control. Visual quality control is facilitated by the incorporation of BrainBrowser–a 3D/4D brain volume viewer (Sherif et al., 2015)–in the web browser-based GUI (**Figure 2**). This makes it possible to visualize the output images of the coregistration, PVC and quantification processing stages without the need for additional software.

**FIGURE 3:**
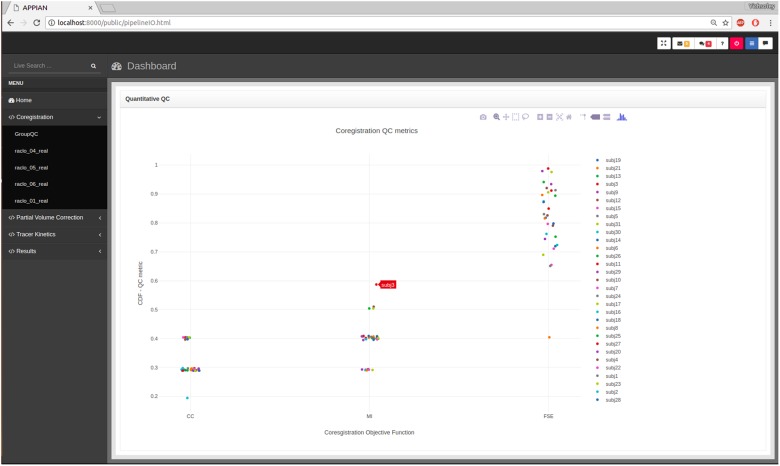
Output from automated quality control (QC) allows users to assess the performance of major processing steps at a glance. Here the automated QC metrics for the coregistration processing stage are shown: CC, cross-correlation; MI, mutual information; FSE, feature-space entropy.

While visual inspection remains the gold-standard method for verifying the accuracy of PET coregistration (Ge et al., 1994; Andersson et al., 1995; Alpert et al., 1996; Mutic et al., 2001; DeLorenzo et al., 2009), automated QC can be useful in guiding the user to potentially failed processing steps. The first stage of the automated QC is to define a QC metric that quantifies the performance of a given processing step. For example, in the case of PET-MRI coregistration the relevant QC metric is the similarity metric that quantifies the joint-dependence of spatial signal intensity distribution of the PET and MR images. By itself a single metric is insufficient to determine whether the processing step has been performed correctly. However, by calculating the distribution of several QC metrics for all subjects, it is possible to identify potential anomalies. Kernel density estimation is used to calculate the probability of observing a given QC metric under the empirical distribution of the entire set of QC metrics. The results are displayed in an interactive plot in the web browser-based dashboard (**Figure 3**).

#### File Formats

Input files for APPIAN are organized following the Brain Imaging Data Structure (BIDS) specifications (Gorgolewski et al., 2016), which uses the Nifti format. In addition, APPIAN also supports input files in the MINC file format (Vincent et al., 2016), which are also organized according to the BIDS specifications but with the MINC file extension.

#### High Performance Computing

APPIAN is optimized for high performance computing in two ways. APPIAN is distributed in a Docker container^3^ that contains all the software necessary to run APPIAN on any computing platform supporting such containers (i.e., where Docker or Singularity has been installed). APPIAN can therefore be run identically across a wide variety of computing environments. This not only facilitates the reproducibility of results, but also allows APPIAN to be deployed simultaneously across multiple computing nodes to analyze subjects in parallel. Additionally, APPIAN supports multithread processing via NiPype and can therefore be run in parallel on multiple CPUs on a given computing platform, e.g., a personal workstation or a processing node on a server.

APPIAN also follows the specification of the BIDS apps in being capable of running subject-level and group-level analyses independently. This means that an instance of APPIAN can be run for each subject in parallel across the available computing resources. Once the individual processing steps have been completed and stored in the same location, the group-level analyses can then be run, e.g., automated QC and reporting of group-level descriptive statistics. Thus, a given data set can be processed with APPIAN at different times and on different computing platforms.

The ability to process large data sets in an easy, fast, and reproducible manner is essential, particularly in cases where parameters for a given algorithm need to be optimized or where the performance of different algorithms at a given processing stage is being compared.

### Accuracy of APPIAN

The accuracy of the APPIAN pipeline was evaluated using the SORTEO Monte-Carlo simulated PET data set (Reilhac et al., 2005). These data consist of 15 subjects with a real T1 MR image segmented into anatomical defined ROIs derived from these images. From each of these anatomically segmented images, three sets of simulated PET images were produced by assigning empirically derived TACs of radiotracer concentrations of [11-C]-raclopride (RCL), [18-F]-fluorodeoxyglucose (FDG), and [18-F]-fluorodopa (FDOPA) into each segmented ROI. The PET images were simulated using the SORTEO Monte-Carlo PET simulator for the Siemens ECAT HR+ scanner (Adam et al., 1997).

Magnetic resonance images were processed using CIVET. CIVET uses the non-parametric N3 method to correct MR field non-uniformity (Sled et al., 1998). The MR image is then transformed to MNI stereotaxic space of the ICBM 152 6th generation non-linear brain atlas (Mazziotta et al., 2001), using a 12 parameter affine transformation (Collins et al., 1994). Spatially normalized images are then segmented into gross anatomical regions with ANIMAL (Collins and Evans, 1997). Thus all ROI images used in the subsequent analysis were derived using CIVET prior to running APPIAN.

The accuracy of the APPIAN was verified by comparing the results of the three central processing stages (coregistration, PVC, quantification) to the true radiotracer concentration TACs or the parametric values derived from them. For the coregistration and PVC stages, the integral of the TAC recovered from the processed images was compared to the integral of the true radiotracer concentration TACs. Parameter values were obtained by calculating the Ki, BPnd, and SUVR for the FDOPA, RCL, and FDG images, respectively, and compared to the same values calculated from the true radiotracer concentration TACs.

The accuracy for each processing stage was calculated by dividing the results from APPIAN by the true radiotracer concentration or parametric values. This calculation was performed for a specific ROI for each radiotracer: cortical GM for FDG, the putamen for FDOPA, and the caudate nucleus for RCL. PVC was performed using the GTM method with a point spread function of 6.5 mm full-width half-maximum (Rousset et al., 1998). The cerebellum was used as a reference region for the calculation of parametric values in the quantification stage.

## Results

**FIGURE 4:**
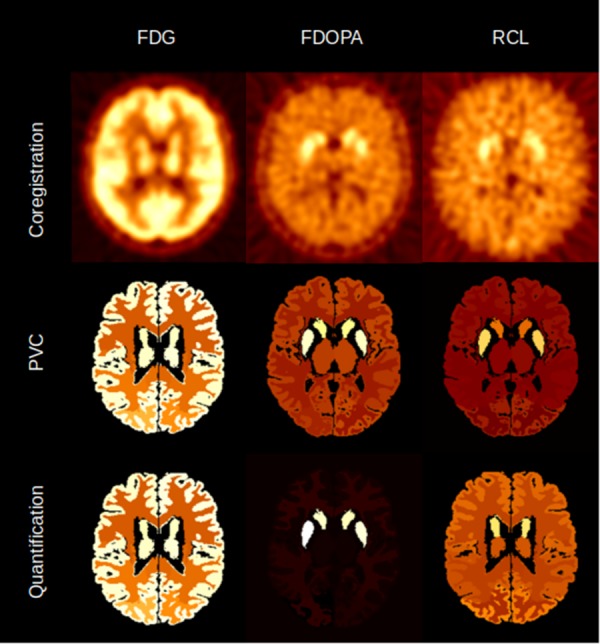
Illustrative example of the image volumes produced by APPIAN for the three major processing stages for FDG, FDOPA, and RCL.

**FIGURE 5:**
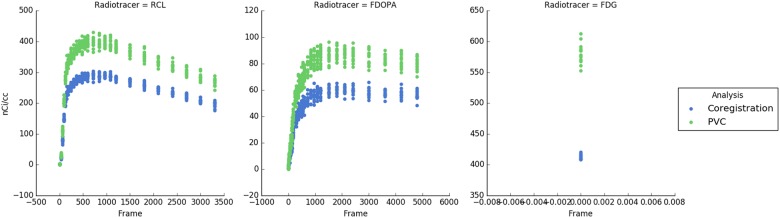
Time-activity curves for each subject and each radiotracer. Blue points indicate the uncorrected PET radioactivity concentration after PET-MRI coregistration and green points show radioactivity concentration after PVC with the GTM method. PVC corrects for spill-over of radiotracer distribution and increases the measured radioactivity concentration.

APPIAN was able to recover accurate values at each major processing stage (**Table 1**), see **Figure 4** for illustrative example from one subject. The recovered values for the coregistration and PVC were the integral of the regional TACs. For the quantification stage the recovered values were the parametric values as described in section “Accuracy of APPIAN”. The accuracy of the coregistration stage was between 0.66 and 0.77, which represented an underestimation of the radiotracer distribution due to partial-volume effects. The accuracy was significantly improved by PVC, ranging between 0.93 and 1.05. The effect of PVC on the uncorrected radioactivity concentration for each radiotracer is shown in **Figure 5**. The PVC led to a slight overestimation in the caudate nucleus with RCL, but near perfect accuracy in the putamen with FDOPA. The final output parametric values were very accurate for RCL (1.02) and FDG (0.94), and lower in the case of FDOPA (0.83).

**Table 1:** Accuracy is measured as the ratio of recovered to true radiotracer concentration or parameter value. APPIAN accurately recovers radiotracer concentrations and tracer kinetic parameters from the SORTEO simulated PET images.

Radiotracer	ROI	PVE	Analysis	Metric	Accuracy
FDG	GM	Uncorrected	Coregistration	integral	0.66 ± 0.006
FDG	GM	Corrected	PVC	integral	0.93 ± 0.025
FDG	GM	Corrected	Quantification	SUVR	0.94 ± 0.048
FDOPA	Putamen	Uncorrected	Coregistration	integral	0.69 ± 0.03
FDOPA	Putamen	Corrected	PVC	integral	1 ± 0.055
FDOPA	Putamen	Corrected	Quantification	Ki	0.83 ± 0.238
RCL	Caudate Nucleus	Uncorrected	Coregistration	integral	0.77 ± 0.016
RCL	Caudate Nucleus	Corrected	PVC	integral	1.05 ± 0.035
RCL	Caudate Nucleus	Corrected	Quantification	BPnd	1.03 ± 0.042


## Discussion

### Accuracy of APPIAN

APPIAN recovered accurate values for each of the three major processing steps on the SORTEO simulated PET data set. Not surprisingly, the accuracy of the recovered parameters was initially low (0.65–0.77), because of partial-volume effects. This improved significantly after PVC with the GTM method (0.93–1.05). For RCL and FDG, the parametric values resulting from the quantification processing stage maintained a similar level of accuracy to that of the PVC radiotracer concentrations. This was not the case with FDOPA where the accuracy decreased from 1 to 0.83. The decrease in accuracy was due to noise in the radiotracer concentrations that were measured in the caudate nucleus, which led to errors in the calculation of the integrals used by the Patlak plot to determine Ki.

For each radiotracer, the validation of APPIAN’s accuracy was performed with differing ROI and using different methods for calculating parametric values. These differences mean that it is not possible to quantitatively compare APPIAN’s accuracy for each radiotracer. The choice of ROI and algorithms for deriving parametric values were chosen to reflect analysis procedures that are widely used by researchers for each of the three radiotracers. It should be noted that the cerebellum is not typically used as a reference region for calculating SUVR or Ki for FDG and FDOPA, respectively. However, while the specific location of the reference region is of utmost importance when performing true PET quantification, it is not relevant for verifying the computational accuracy of the algorithms in the APPIAN pipeline.

### Comparison to Existing Pipelines

Several PET processing pipelines have been presented in recent years. We here briefly describe them to highlight their relative strengths (**Table 2**) and discuss how APPIAN compares to these. There are other PET pipelines that carry out at least three of the six steps performed by APPIAN, they are: PMOD (Mikolajczyk et al., 1998), CapAIBL (Bourgeat et al., 2015), MIAKAT (Gunn et al., 2016), Pypes (Savio et al., 2017), and NiftyPET (Markiewicz et al., 2017).

**Table 2:** Many different PET processing software exist with various features.

Feature	MIAKAT	PMOD	Pypes	CapAIBL	NiftyPET	APPIAN
Cost	Free	2,970–14,850$	Free	Free	Free	Free
Open-source	Yes	No	Yes	No	Yes	Yes
Language	MATLAB	Java	Agnostic^∗^	C++	Python	Agnostic^∗^
Quantification	Yes	Yes	No	SUVR	No	Yes
PVC	No	No	Yes	No	Yes	Yes
Structural imaging	Yes	Optional	Yes	No	Yes	Required
Cloud-based processing	No	DICOM server	No	Yes	Maybe	Yes
Local processing	Yes	Yes	Yes	No	Yes	Yes
Visualization	GUI	GUI	Result plots	3D surfaces	No	Dashboard
Surface-based	No	No	No	Yes	No	Yes
Reconstruction	No	No	No	No	Yes	No


*APPIAN attempts to provide all post-reconstruction tools needed for PET research. ^∗^Agnostic: these packages are written in Python but support software written in any language as long as it can run on the command line. Here, some of the most established and more recent pipelines are compared to APPIAN.*

#### PMOD

PMOD (Mikolajczyk et al., 1998) is the gold-standard software for quantification of PET images and is distributed in modules that perform specific aspects of PET analysis. PKIN includes an exhaustive list of quantification models and preprocessing methods for blood and plasma activity curves for analyzing regional PET data, while PXMOD performs the same analyses at the pixel level. PMOD also has modules that perform analysis and PVC (PBAS), and image registration (PFUS). All these modules can be used interactively using a graphical user interface (GUI) but can also be linked together in a pipeline to automate the analysis of large data sets. A particularly useful feature is the option to add a QC step after each processing stage. PMOD thus includes all the preprocessing and analysis methods needed for automated PET analysis. As a commercial software solution however, the PMOD code is not open-source and thus imposes limitations on the user community with respect to flexible development and implementation of new image processing and analytical methods.

#### CapAIBL

CapAIBL (Bourgeat et al., 2015) is a surface-based PET processing pipeline that is available through an online platform. It spatially normalizes PET images to cortical surface templates for the surface-based analysis and visualization of PET data without the need for structural imaging. Cortical surfaces are derived from a standardized template, thus subcortical structures such as the basal ganglia are not included in the analysis. A purely surface-based approach is also limited to images from structurally intact brains and may thus be difficult to apply to datasets with focal brain lesions. Nonetheless, CapAIBL provides a highly original method for performing automated PET analysis that is useful for the study of the cerebral cortex in cases where no structural image has been acquired alongside the PET image. Dore et al. (2016) have shown a close correspondence in PET quantification across a wide range of radiotracers with coregistered PET and MR images and using CapAIBL, i.e., without coregistration.

#### Pypes

A recent multi-modal pipeline, Pypes (Savio et al., 2017), combines PET analysis with structural, diffusion, and functional MR images. This pipeline is free, open-source, and it is also written using NiPype (Gorgolewski et al., 2011). Pypes leverages several brain imaging software packages–including SPM12 (Ashburner, 2012), FSL (Jenkinson et al., 2012), and AFNI (Cox, 2012)–to provide multi-modal workflows. While Pypes does incorporate PVC, it does not incorporate tracer kinetic analysis, flexible ROI definition, or automated QC.

#### MIAKAT

MIAKAT (Mikolajczyk et al., 1998) is the most complete, open-source PET processing pipeline. In addition to featuring many tracer-kinetic models, MIAKAT also includes motion-correction; a feature that is not currently implemented in APPIAN. One of MIAKAT’s most important features is its user-friendly GUI. This makes MIAKAT easy to use for users not familiar with the command-line interface. In addition to analyzing PET images, MIAKAT also includes the option to include structural images which are used to define regions of interest (ROI). MIAKAT has been recently extended for use on non-brain PET image analysis and for application to species other than humans (Searle and Gunn, 2017).

One limitation of MIAKAT is that it does not include PVC, although this could potentially be added to the pipeline. More importantly, it is built using MATLAB, which restricts MIAKAT to a single, proprietary language with licensing restrictions.

#### NiftyPET

NiftyPET is another open-source, Python-based PET processing pipeline that implements Graphical Processing Unit-processing for massively parallel processing (Markiewicz et al., 2017). It is the only PET processing pipeline to reconstruct PET images from sinograms and to perform PVC (Yang et al., 1995). It should be noted that the authors of NiftyPET use the term “quantification” to refer to quantification of radioactivity concentrations, whereas this term is here used to refer to the quantification of underlying biological or physiological parameters. NiftyPET therefore does not include parametric quantification.

#### APPIAN

**FIGURE 6:**
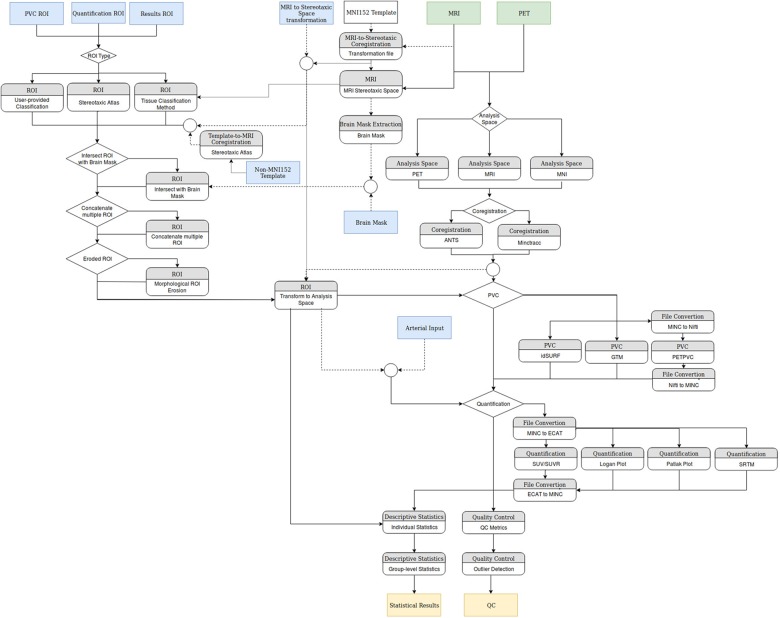
Flowchart of the modules implemented in APPIAN. Green boxes indicate mandatory inputs, blue boxes indicate optional inputs, and tan boxes indicate the primary quantitative outputs of the pipeline.

There are a wide variety of PET pipelines presently available, each satisfying a different niche. APPIAN provides a highly flexible framework for processing large PET data sets, see **Figure 6** for a detailed flowchart of APPIAN. One important feature is that APPIAN allows the user to define ROI from a variety of sources and is therefore compatible with a wide variety of experimental designs. Whereas lesion studies frequently use a binary lesion image defined on each subject’s respective structural image in its native coordinate space, it may be necessary for some studies (e.g., investigating lesion effects on functional systems as in aphasia post stroke) to use a common brain atlas in MNI-space. On the other hand, PET studies of, e.g., microglial inflammation may identify ROI based on the subjects’ respective tracer binding pattern in PET images in their native space. Quantification of PET images also requires users to be able to use either ROI to define a reference region without specific binding of the radiotracer or TAC measured from arterial blood samples. APPIAN is therefore suited for a wide variety of experimental contexts because of its flexible system for ROI definition.

APPIAN is also modular and easily extendable so that users can either test new algorithms, e.g., a new PVC method, or add entirely new analyses to the pipeline. Moreover APPIAN, like Pypes, is written with NiPype and can thus use any program that can be run in a Bash shell environment. Users therefore do not need to rewrite their software in, e.g., Python if they wish to implement it in APPIAN. Also, given that descriptive statistics for ROI are automatically generated in the reporting stage, it is easy to extend APPIAN to perform sophisticated group-wise analyses. For example, investigators interested in implementing graph theoretical analyses can append their analysis to the group-level processing and input the descriptive statistics that are collected at the reports stage to their analysis.

Finally, APPIAN implements automated and visual QC to facilitate the analysis of large data sets. This is essential because as multiple processing stages are linked together into increasingly sophisticated pipelines, it is important that users be able to easily and reliably confirm that each processing stage has been performed correctly.

### Using APPIAN

APPIAN is available for both local use and cloud-based use. The source code for APPIAN is freely available^4^. While the code-base will be maintained by the authors, we hope to create a community of developers to support the project in the long-term. Changes to APPIAN will be validated against the open CIMBI PET data^5^ (Knudsen et al., 2016). APPIAN is provided via a Docker (see footnote 3) image and can be easily downloaded from Docker hub under tffunck/appian:latest. Cloud-based APPIAN is available via the CBRAIN platform^6^.

## Conclusion

APPIAN is a novel PET processing pipeline that seeks to automate the processing of reconstructed PET images for a wide variety of experimental designs. It is therefore flexible and easily extendable. In order to ensure that each processing step is performed as expected, visual and automated QC are implemented. Our results on Monte-Carlo simulated PET data have shown that APPIAN accurately recovers radiotracer concentration and parametric values. Future work will focus on increasing the sensitivity of the automated QC and implementing more algorithms for coregistration, PVC, and quantification.

## Author Contributions

TF is the primary author of the manuscript, developed the APPIAN code. KL developed the APPIAN code. P-JT is the advisor for designing APPIAN, edited the manuscript, and ongoing development of new PET quantification models. AT (principal investigator) and AE (co-principal investigator) provided conceptual guidance and edited the manuscript.

## Conflict of Interest Statement

KL was employed by the company Biospective, Inc. and AE is founder and director of the company Biospective, Inc. The remaining authors declare that the research was conducted in the absence of any commercial or financial relationships that could be construed as a potential conflict of interest. The reviewer JP and handling Editor declared their shared affiliation.

## Footnotes

1 https://github.com/BIC-MNI/minc-toolkit-v2

2 http://www.turkupetcentre.net/petanalysis/format_tpc_dft.html

3 https://www.docker.com/

4 www.github.com/APPIAN-PET/APPIAN

5 https://openneuro.org/datasets/ds001421

6 http://portal.cbrain.mcgill.ca/
